# A Large-Scale Dataset and Deep Learning Model for Detecting and Counting Olive Trees in Satellite Imagery

**DOI:** 10.1155/2022/1549842

**Published:** 2022-01-15

**Authors:** Amr Abozeid, Rayan Alanazi, Ahmed Elhadad, Ahmed I. Taloba, Rasha M. Abd El-Aziz

**Affiliations:** Department of Computer Science, College of Science and Arts in Qurayyat, Jouf University, Sakakah, Saudi Arabia

## Abstract

Since the Pre-Roman era, olive trees have a significant economic and cultural value. In 2019, the Al-Jouf region, in the north of the Kingdom of Saudi Arabia, gained a global presence by entering the Guinness World Records, with the largest number of olive trees in the world. Olive tree detecting and counting from a given satellite image are a significant and difficult computer vision problem. Because olive farms are spread out over a large area, manually counting the trees is impossible. Moreover, accurate automatic detection and counting of olive trees in satellite images have many challenges such as scale variations, weather changes, perspective distortions, and orientation changes. Another problem is the lack of a standard database of olive trees available for deep learning applications. To address these problems, we first build a large-scale olive dataset dedicated to deep learning research and applications. The dataset consists of 230 RGB images collected over the territory of Al-Jouf, KSA. We then propose an efficient deep learning model (SwinTUnet) for detecting and counting olive trees from satellite imagery. The proposed SwinTUnet is a Unet-like network which consists of an encoder, a decoder, and skip connections. Swin Transformer block is the fundamental unit of SwinTUnet to learn local and global semantic information. The results of an experimental study on the proposed dataset show that the SwinTUnet model outperforms the related studies in terms of overall detection with a 0.94% estimation error.

## 1. Introduction

Nowadays, the availability of satellite services is establishing a lot of promising research and applications, for example, detecting, locating, and counting objects (such as people, vehicles, buildings, and farms), monitoring, 3D reconstructions, and geographical analysis [1].

In the computer vision field, object counting is a well-known problem that aims to figure out how many objects are in a static image or video frame. Object counting is an active research field and has many use cases in diverse domains, such as ecologic studies, crowd counting, microcell counting, and vehicle counting [2, 3].

In traditional methods, handcrafted features (e.g., SIFT [4] and HOG [5]) are extracted to detect and count olive trees from a stationary image. Unfortunately, the performance of these traditional methods is affected by many factors such as scale variations, weather changes, perspective distortions, and orientation changes. Recently, deep learning detection models such as single shot multibox detector (SSD) [6] and region convolutional neural network (R-CNN) [7] achieve high performance and provide a promising solution for these challenges [3]. Despite the successes of deep learning methods, there is a lack of a standard database of olive trees available for deep learning applications.

Therefore, we first created a large-scale olive dataset for deep learning research and applications. The dataset consists of 230 RGB satellite imagery collected across Al-Jouf, Saudi Arabia. The images have been obtained from Satellites Pro [8], which provides satellite imagery and maps for most countries and cities around the world. To lighten the workload and expedite the annotation process, the olive trees are labeled with center points. The proposed dataset is useful for many olive tree deep learning applications such as detection, counting, and segmentation.

Then, inspired by the success of the Swin Transformer [9], we propose an efficient deep learning model (SwinTUnet) for detecting and counting olive trees. SwinTUnet is a Unet-like network that includes an encoder, a decoder, and skip connections. Instead of using the convolution operation, Swin Transformer is used to learn better local and global semantic information. The proposed SwinTUnet model outperforms the related studies in terms of overall detection, with a 0.94 percent estimation error, according to the results of an experimental study on the proposed dataset.

The structure of this paper is as follows: Section 2 presents a related work. The proposed SwinTUnet architecture is explained and discussed in Section 3.  Section 4 discusses the experiments and their outcomes. Finally, in Section 5, the conclusion and future work are presented.

## 2. Related Work

The use of remote sensing to automatically identify trees was first used for forestry applications [10]. Tree detection and enumeration in crop fields have always been a priority for the research community in recent years. There are numerous techniques available to effectively solve the problem of identifying and counting olive trees. Based on image processing methods, olive tree identifying and counting from satellite and UAS images can, generally, be classified into three groups.

### 2.1. Classification Methods

In these methods, image classification methods are used to classify and then count the olive trees. Bazi et al. [11] adapted the Gaussian process classifier (GPC) and the erosion morphological filter [12] to automatically counting olive trees in a selected zone of Saudi Arabia. The algorithm detected 1124 trees out of 1167 with a 96% overall detection rate. Land cover classes contain nonolive trees, buildings, and ground. Olive trees were counted using blob analysis and classified into land cover classes. Despite the high level of overall accuracy, the classifier was trained using a smaller number of training samples, so there was room for improvement.

Moreno-Garcia et al. [13] proposed an approach based on the fuzzy logic to classify and then count olive trees in very high resolution (VHR) images using the *k*-neighbor approach. This method was tested using RGB satellite imagery obtained from the SIGPAC viewer covering an area of Spain. With a 1-in-6 omission rate and zero omission rate, the results were promising, but the number of testing images and diversity were insufficient.

Peters et al. [14] applied an object-based feature extraction and classification for olive landscape mapping based on VHR from various sensors (optical and radar data). A four-step model was used to detect olive trees across the French countryside. This model consists of segmentation, extraction of features, classification, and, finally, mapping of results. Synergy methods were applied at each phase by merging features from numerous sensors. As a result, the overall accuracy was 84.3%.

### 2.2. Segmentation Methods

Image segmentation is the operation of partitioning the input image to extract a region of interest (ROI) that holds important information. Many segmentation methods (such as edge detection, thresholding, region growing, and clustering) were used to identify and count olive trees. These methods were individually proposed as well as a hybrid approach.

Moreno-Garcia et al. introduced a technique to identify and segment trees of olive using the *K*-means algorithm from satellite imagery [15]. This technique was quick with fewer clusters and could tell the difference between ground and satellite data. This methodology yielded a 0% omission error rate and a 1-in-6 commission error rate. Despite the marked results, the number and diversity of the images tested were insufficient. Waleed et al. used the improved *K*-means to count trees of olive across large areas [16]. Some of the steps in the technique include preprocessing, image segmentation, extraction of features, and classification. For the purpose of segmenting the region of interest (ROI), *K*-means segmentation was used. The development of various classifiers yielded promising results. With a training and testing accuracy of 97.5 percent, the random forest classification technique produces good results.

Another technique applied a thresholding strategy with different levels to segment and extract olive trees from the foreground [17]. They proposed a robust and an efficient model for accurately segmenting and detecting olive trees in a variety of environments. This technique yielded promising results, with a 96 percent overall accuracy. However, the existence of nonolive elements in the total count left some room for improvement.

In [2], the authors presented a color-based segmentation application for olive tree counting from images acquired from unmanned aerial systems (UASs) and utilized a cloud service. The application produced promising results, 330 of 332 trees were counted, but the latency and computational time were not overcome by a mixture of on-board processing and cloud-based services.

### 2.3. Detection Methods

Aerial views of trees reveal morphological features that resemble blobs. These blobs appear brighter at the tips when viewed from above, with shadows following them to their base. The Laplace operator, also known as the Laplacian, is a differential operator in the Euclidean space defined by the divergence of a function's gradient. The Laplacian operator is primarily used for edge and blob detection [1].

For the detection of the olive, Karantzalos and Argialas used Laplacian spatial resolution with local maximum points [18]. Satellite greyscale images from the QuickBird and IKONOS satellites were used to test their algorithm. Blob detection is a popular technique because of its simplicity and reliability; however, it is vulnerable to missing olive data. The technique only used trees with circular morphology and treated each object with those characteristics as the olive tree.

Daliakopoulos et al. introduced a hybrid method between the Arbor crown enumerator (ACE) and the Laplacian of Gaussian (LOG) to detect olive trees as blobs for VHR satellite images [19]. The method used red band thresholding and blob detection based on the Normalized Difference in Vegetation Index (NDVI) [20]. The hybrid method overcomes the disadvantages of separately thresholding and blob detection. With an estimation error of 1.3%, accurate detection and count were observed. However, with high computational cost, the algorithm produced accurate results.

Waleed et al. [1] proposed a multistep technique to identify and count olive trees. This technique consists of multiple image optimization and edge detection steps. The information of the red band was extracted from RGB images acquired using the SIGPAC viewer. The single red band is sharpened, and edges are detected after it is extracted. Using morphological reconstruction, the closed edges formed by tree boundaries are transformed into white blobs. With an estimation error of 1.27%, results were generated over a variety of images capturing ground truth information.

The use of various techniques for the automatic detecting and counting of olive trees has been documented in the literature. Simple and effective techniques such as image segmentation, as well as training and testing samples, all outperformed complex classifiers. However, it was discovered that, as the image information was increased, the accuracy improved. Although the traditional method has achieved high accuracy, it is not stable and is affected by many satellite challenges, such as variation of the viewpoint, image scale, quality, and orientation. Furthermore, the datasets used lacked the necessary diversity in terms of number and ground classes.

Despite the success of the above handcrafted feature methods, many factors such as scale variations, weather changes, perspective distortions, and orientation changes affect the performance of these traditional methods. Deep learning models, such as [2, 6, 7], have recently achieved high performance and offer a promising solution to these problems. However, the lack of a standard dataset for olive farms is a major impediment to deep learning techniques being used in this field. Therefore, we begin by creating a large-scale olive dataset for deep learning research and applications. The dataset consists of 230 RGB images collected across Al-Jouf, Saudi Arabia. Then, we propose an efficient deep learning model (SwinTUnet) for detecting and counting olive trees.

## 3. The Proposed Model

**Figure 1:**
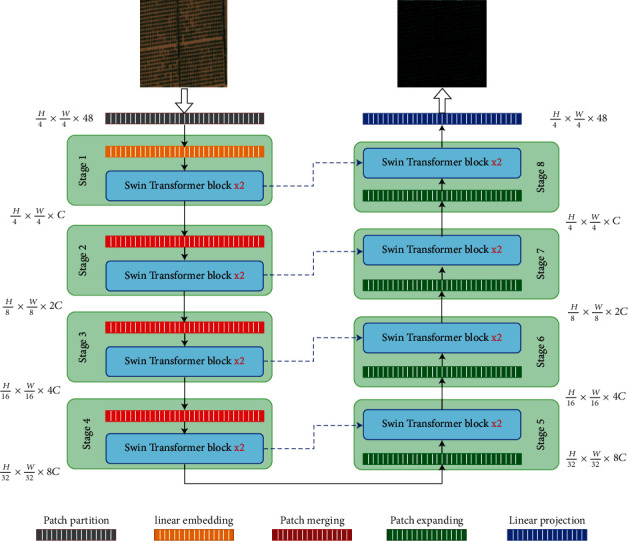
The architecture of the SwinTUnet model includes encoders, decoders, and skip connections. The Swin Transformer is the base of building the model.

Figure 1 depicts the proposed SwinTUnet architecture, which includes an encoder, a decoder, and skip connections. Swin Transformer block [9] is the fundamental unit of SwinTUnet. The encoder is used to make a series of embeddings out of the inputs. The olive satellite images are divided into 4 × 4 nonoverlapping patches. Based on this partitioning method, each patch now has a feature dimension of 4 × 4 × 3 = 48. The projected feature dimension is also converted into an arbitrary dimension using a linear embedding layer (represented as C).

The hierarchical feature representations are created by passing the tokens (transformed patches) across several blocks of Swin Transformer and layers of patch merging. Downsampling and increasing dimension are handled by the patch merging layer, while feature representation learning is handled by the Swin Transformer block. We create a symmetric transformer-based decoder, which is inspired by U-net [21]. The decoder is constructed from Swin Transformer blocks and the opposite patch expanding layers. The derived context features are merged with multiresolution features out from the encoder through the use of skip connections to cover the loss of the spatial features due to downsampling.

A patch expanding layer, unlike a patch merging layer, is specifically applied for upsampling the size of features. The layer of patch expanding resizes adjacent-dimension feature vectors into large feature vectors with upsampling the resolution by two. Finally, the final patch expanding layer is applied to the feature maps to perform four upsamplings of the resolution to the original resolution (*W* and *H*). Then, on top of these upsampled features, a linear projection layer is used to create the density map.

### 3.1. Swin Transformer Block

**Figure 2:**
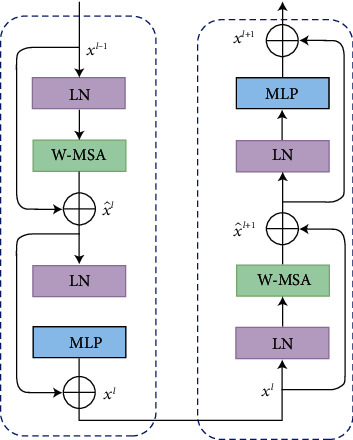
Two sequential Swin Transformer blocks.

Unlike the traditional multihead self-attention (MSA) module, the Swin Transformer block [9] is based on the use of shifted windows.  Figure 2 depicts each Swin Transformer block which includes a LayerNorm (LN) layer, a MSA module, a residual connection, and two MLP layers. In the two successive transformer blocks, the window-based MSA (W-MSA) and shifted window-based MSA (SW-MSA) modules are used. Using a window partitioning mechanism, sequential Swin Transformer blocks can be formulated as follows:

(1) $$\begin{array}{c} {\hat{x}}^{l}=\text{W}-\text{MSA}\left(LN\left(x^{l-1}\right)\right)+\;x^{l-1},\\ x^{l}=\text{MLP}\left(LN\left({\hat{x}}^{l}\right)\right)+\;{\hat{x}}^{l},\\ {\hat{x}}^{l+1}=\text{SW}-\text{MSA}\left(LN\left(x^{l}\right)\right)+\;x^{l},\\ x^{l+1}=\text{MLP}\left(LN\left({\hat{x}}^{l+1}\right)\right)+\;{\hat{x}}^{l+1}, \end{array}$$

where ${\hat{x}}^{l}$ and *x*^*l*^ are the output features of the *l*^*th*^ block, (S)W-MSA and MLP modules, respectively. The MSA is calculated in the same way as in previous research [22, 23]:

(2) $$\begin{array}{c} \text{MSA}\left(\text{Q,K,V}\right)=\text{softMax}\left(\frac{QK^{T}}{\sqrt{d}}+\text{Bias}\right)V, \end{array}$$

where *Q*,  *K*,  *V*  ∈  *ℝ*^*N*^2^×*d*^ represent the query, key, and value matrices. Respectively, *d* and *N*^2^ denote the query or key dimension and the patch number in a window. The bias values are extracted from the matrix of bias $\hat{B}\in \;{\mathbb{R}}^{\left(2N-1\right)\times \left(2N+1\right)}$.

### 3.2. Encoder

During the encoder, two sequential Swin Transformer blocks are applied on the input tokens with a resolution of *H*/4 × *W*/4 and 48 dimensions to produce representation learning. The output resolution and feature dimension were left unchanged. As the network expands, the token number is reduced to produce a hierarchical representation by patch merging layers. The first patch merging layer merges the features of each group of 2 × 2 adjacent patches. After that, a linear layer is applied to the merge features in 4*C* dimensions. The output dimension is set to 2*C*, and the token number is reduced by 2 × 2 = 4. Following this, Swin Transformer blocks are utilized to transform the features, keeping the resolution at *H*/8 × *W*/8. Stage 2 refers to the first section of patch merging and feature transformation. The procedure is repeated twice more, with different output resolutions with the size of *H*/16 × *W*/16 and *H*/32 × *W*/32, respectively, as “stage 3” and “stage 4.” The four stages are enough to learn the deep feature representation because the transformer is too deep to be converged [24].

### 3.3. Decoder

Swin Transformer block is the backbone of both encoder and decoder. Unlike to the encoder, the patch expanding layer is used in the decoder instead of the patch merging layer to upsample the constructed features. By reshaping neighboring dimension feature maps and reducing the dimension of the feature by 2 of its input dimensions, the patch expanding layer increases the resolution of the feature map. Consider the first patch expanding layer; before upsampling, a linear layer is utilized to double the dimension of the feature (*H*/32 × *W*/32  × 8*C*) to be (*H*/32 × *W*/32  × 16*C*). Then, we use the rearrange operation to double the resolution and reduce the dimension of the feature to a quarter of its original size (*H*/32 × *W*/32  × 16*C*⟶*H*/32 × *W*/32  × 4*C*.

The skip connections, like the U-net [21], are used to inject the upsampled features by the multiscale features from the encoder. To reduce the spatial feature loss caused by downsampling, we concatenate the shallow and deep features together. The concatenated features' size is kept like the upsampled features' size after a linear layer.

### 3.4. Implementation Details

In the implementation, we used a well-known PyTorch [25] library. Then, the proposed model was trained and tested on an NVIDIA GeForce RTX 2060 GPU. Before training, random data augmentations such as rotating, scaling, and flipping are used to raise a data variety. The input image was resized into 224 × 224 which leads to overcome the problem of GPU out of memory in the training. The model parameters are set using the weights pretrained on ImageNet-1K [26]. During the training period, our model is optimized for backpropagation using the well-known SGD optimizer [27] with momentum value 0.9 and weight decay equal to 1*e*^−4^.

## 4. Experiments and Discussion

This section discusses the proposed olive tree dataset used to evaluate our model. It also covers the metrics that were used to assess the proposed model's performance.

### 4.1. Dataset

**Figure 3:**
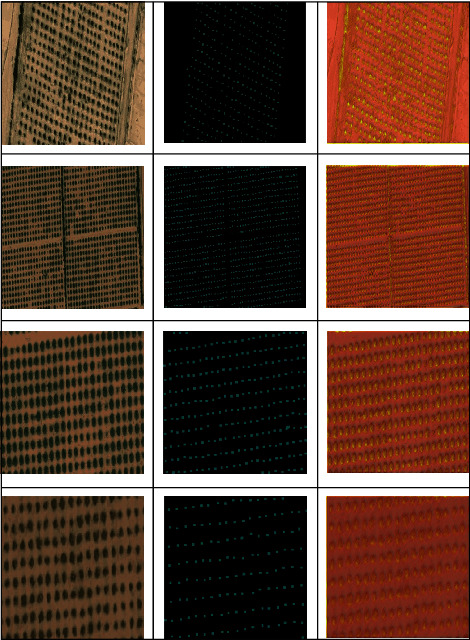
Sample olive images (the first column shows original images, the second column shows ground truth, while the third column shows corresponding detection results).

According to the statistics of the Ministry of Environment and Water Branch in Al-Jouf, 2019, the Al-Jouf region hosts 30 million trees, foremost among which are olive trees (18 million trees), which annually produce 10 thousand tons of oil [8, 9]. Therefore, our dataset consists of 230 images gathered from Al-Jouf, KSA territory, by using the Satellites Pro. The Satellites Pro provides satellite imagery and maps over most countries and cities of the world. The RGB images of size 512 × 512 were taken of the target area having 32 bit of information. Some image samples are presented in Figure 3.

The olive trees are marked with center points to reduce workload and accelerate annotation. In the first step, olive images are labeled by bounding boxes enclosing the olive trees. The four vertices of the bounding boxes are denoted as {(*x*_*i*_,  *y*_*i*_),   *i* = 1,  2,  3,  4}. In the second step, we calculate the centroid of each box as the central location by the following formula:

(3) $$\begin{array}{c} \left(x,y\right)=\left(\frac{1}{4}\sum_{i=1}^{4}x_{i},\frac{1}{4}\sum_{i=1}^{4}y_{i}\right). \end{array}$$

### 4.2. Evaluation Metrics

To evaluate different techniques on different datasets, the performance of our model was evaluated using a variety of performance metrics.

(1) Overall accuracy (OA): it is the percentage of olive trees correctly identified out of the actual total olive tree number. It displays correctly identified number of trees in the ground truth data among the marked ones. Overall accuracy is calculated mathematically using the following equation:
  (4) $$\begin{array}{c} OA=\frac{NE}{NA}\;\times 100, \end{array}$$
where *NE* represents the estimated olive tree number and *NA* is the actual olive tree number.
(2) Omission error rate (OER): it is the percentage of positive test subjects who are misidentified as negative test subjects. In other words, OER is the percentage of times our proposed system fails to recognize olive trees as such. OER is calculated mathematically using the following equation:
  (5) $$\begin{array}{c} \text{OER}=\frac{Nm}{NA}\;\times 100, \end{array}$$
where *Nm* denotes the number of omitted olive trees.
(3) Commission error rate (CER): it is defined as the presence of negative samples that have been mistakenly identified as positive. It happens when there are nonolive trees in the output. CER is calculated mathematically using the following equation:
  (6) $$\begin{array}{c} \text{CER}=\frac{Nf}{NA}\times 100, \end{array}$$
where *Nf* denotes the number of false trees detected.
(4) Estimation error (EE): it refers to the difference between the identified number of objects and the number of objects to be identified. In our proposed model, it is the difference between an actual and estimated number of olive trees in the sample divided by the actual number of olive trees. EE is calculated mathematically using the following equation:
  (7) $$\begin{array}{c} EE=\frac{NE-NA}{NA}\;\times 100. \end{array}$$

### 4.3. Overall Evaluation of the Proposed Model

Based on evaluating the proposed model, the overall estimation error for testing was 0.94%. As shown in Table 1, for a 100% distribution of olive and nonolive trees along with other objects, about 0.97% of nonolive data was miscalculated as olive, and 1.2% of olive data was miscalculated as nonolive. The results of testing on the proposed dataset showed an overall identification with a 0.94% estimation error.

Figure 3 depicts an olive image along with ground truth and corresponding detection results. The image depicts a mix of distributions of olive trees with notable distance between them and those that are closely planted. Our proposed model correctly identified almost all the olive trees, but it miscounted the young and closely planted trees.

### 4.4. Comparative Analysis with Related Work

The results of the proposed model were compared to those of existing olive detection and counting techniques. The dataset's parameters, the processed number of images, the spectrum representing the size of processed data, and evaluated performance were all used in the comparison.  Table 2 demonstrates the results of a comparison of the proposed model to existing methods.

**Figure 4:**
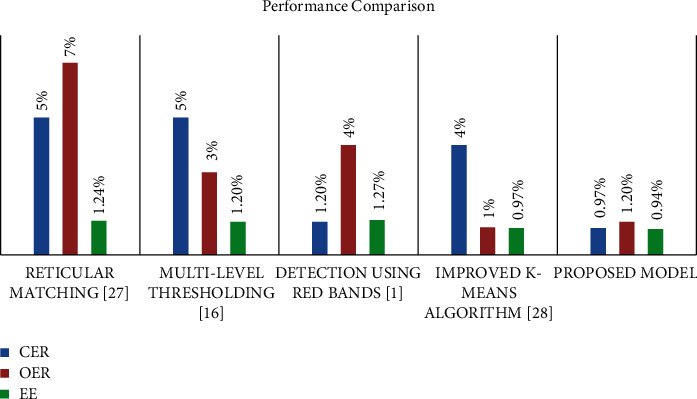
Performance comparison of the proposed model and the existing work.

As shown in Figure 4, our proposed model was evaluated on a large-scale dataset and yielded high accuracy, indicating that our model is accurate and robust. Our proposed model addressed the flaws in related techniques by accurately identifying and counting olive trees. This novel model of olive tree detecting and counting was validated over RGB images with an overall accuracy of 98.3%, which overcomes related work. Testing on the large-scale dataset which consists of olive trees and other ground objects, the proposed model had the lowest overall estimation error of 0.94% of the existing techniques. It is worth noting that our proposed dataset includes 230 images of both olive trees and other objects.

## 5. Conclusion

In conclusion, we have proposed an effective deep learning model (SwinTUnet) for detecting and counting olive trees from satellite imagery. SwinTUnet is a Unet-like network that includes an encoder, a decoder, and skip connections. Instead of using the convolution operation, the SwinTUnet adapted the Swin Transformer block to learn local and global semantic information. Moreover, we started by constructing a large-scale olive dataset for deep learning research and applications. The dataset consists of 230 RGB images collected across Al-Jouf, Saudi Arabia. According to the results of an experimental study on the proposed dataset, the SwinTUnet model outperforms the related studies in terms of overall detection, with a 0.94% estimation error.

However, there are some drawbacks, such as the difficulty in identifying olive trees that are close to other trees. Consequently, in the future, we plan to extend the proposed dataset by more images from various sources and enhance the proposed model.

## Figures and Tables

**Table 1:** Overall evaluation of the proposed model.

No. of images	Total trees	Detected trees	EE (%)	CER	OER
230	73286	72598	0.94	709 (0.97%)	912 (1.2%)

**Table 2:** A comparison of the proposed model's results to previous research.

Technique	Dataset	Spectrum	No. of images	Performance metrics			
				OA	CER (%)	OER (%)	EE (%)
Reticular matching [28]	QuickBird	Greyscale	N/A	98%	5	7	1.24
Multilevel thresholding [17]	SIGPAC viewer	Greyscale	95	96%	5	3	1.2
Detection using red bands [1]	SIGPAC viewer	Red band	60	N/A	1.2	4	1.27
Improved *K*-means algorithm [16]	SIGPAC viewer	RGB	110	97.5%	4	1	0.97
Proposed model	Satellites Pro	RGB	230	98.3%	0.97	1.2	0.94

## Data Availability

The data that support the ﬁndings of this study are available from the corresponding author upon reasonable request.

## References

[1] Waleed M., Um T.-W., Khan A., Ahmad Z. (2020). An automated method for detection and enumeration of olive trees through remote sensing. *IEEE Access*.

[2] Gao G., Liu Q., Wang Y., Sensing R. (2020). Counting from sky: a large-scale data set for remote sensing object counting and a benchmark method. *IEEE Transactions on Geoscience and Remote Sensing*.

[3] Gao G., Liu Q., Wen Q., Wang Y. (2020). PSCNet: Pyramidal Scale and Global Context Guided Network for Crowd Counting. https://arxiv.org/abs/2012.03597.

[4] Eldahshan K., Farouk H., Abozeid A., Eissa M., Technology A. I. (2019). Global dominant SIFT for video indexing and retrieval. *Journal of Theoretical and Applied Information Technology*.

[5] Dalal N., Triggs B. Histograms of oriented gradients for human detection.

[6] Liu W., Anguelov D., Erhan D. Ssd: single shot multibox detector.

[7] Ren S., He K., Girshick R., Sun J., intelligence m. (2016). Faster R-CNN: towards real-time object detection with region proposal networks. *International Journal of Geographical Information Science*.

[8] Pro S. (2021). Satellite World Maps and Imagery. https://satellites.pro/.

[9] Liu Z., Lin Y., Cao Y. (2021). Swin Transformer: Hierarchical Vision Transformer Using Shifted Windows. https://arxiv.org/abs/2103.14030.

[10] Zhen Z., Quackenbush L., Zhang L. (2016). Trends in automatic individual tree crown detection and delineation-evolution of LiDAR data. *Remote Sensing*.

[11] Bazi Y., Al-Sharari H., Melgani F. An automatic method for counting olive trees in very high spatial remote sensing images.

[12] Jankowski M. Maine, USA, “Erosion, dilation and related operators.

[13] Moreno-Garcia J., Jimenez L., Rodriguez-Benitez L., Solana-Cipres C. J. Fuzzy logic applied to detect olive trees in high resolution images.

[14] Peters J., Van Coillie F., Westra T., De Wulf R. (2011). Synergy of very high resolution optical and radar data for object-based olive grove mapping. *International Journal of Geographical Information Science*.

[15] Moreno-Garcia J., Linares L. J., Rodriguez-Benitez L., Solana-Cipres C. Olive trees detection in very high resolution images.

[16] Waleed M., Um T.-W., Khan A., Khan U. (2020). Automatic detection system of olive trees using improved K-means algorithm. *Remote Sensing*.

[17] Khan A., Khan U., Waleed M. (2018). Remote sensing: an automated methodology for olive tree detection and counting in satellite images. *IEEE Access*.

[18] Karantzalos K., Argialas D. (2004). Towards automatic olive tree extraction from satellite imagery. *Geo-Imagery Bridging Continents. XXth ISPRS Congress*.

[19] Daliakopoulos I. N., Grillakis E. G., Koutroulis A. G., Tsanis I. K., Sensing R. (2009). Tree crown detection on multispectral VHR satellite imagery. *Photogrammetric Engineering & Remote Sensing*.

[20] Filippa G., Cremonese E., Migliavacca M. (2018). NDVI derived from near-infrared-enabled digital cameras: applicability across different plant functional types. *Agricultural and Forest Meteorology*.

[21] Ronneberger O., Fischer P., Brox T. U-net: Convolutional networks for biomedical image segmentation.

[22] Hu H., Gu J., Zhang Z., Dai J., Wei Y. Relation networks for object detection.

[23] Hu H., Zhang Z., Xie Z., Lin S. Local relation networks for image recognition.

[24] Touvron H., Cord M., Sablayrolles A., Synnaeve G., Jégou H. (2021). Going Deeper with Image Transformers. https://arxiv.org/abs/2103.17239.

[25] Paszke A., Gross S., Massa F., Lerer A. (2019). Pytorch: an imperative style. *High-performance Deep Learning Library*.

[26] Deng J., Dong W., Socher R., Li L.-J., Li K., Fei-Fei L. Imagenet: a large-scale hierarchical image database.

[27] Duda J. (2019). SGD Momentum Optimizer with Step Estimation by Online Parabola Model. https://arxiv.org/abs/1907.07063.

[28] González J., Galindo C., Arevalo V., Ambrosio G. Applying image analysis and probabilistic techniques for counting olive trees in high-resolution satellite images.

